# Clampless off-pump coronary artery bypass prevents early post-operative neurologic injury compared to on-pump: a propensity score matched analysis

**DOI:** 10.1186/1749-8090-8-S1-O170

**Published:** 2013-09-11

**Authors:** C Bassano, F Uva, E Bovio, R Scaini, L Chiariello

**Affiliations:** 1 Cardiac Surgery, Tor Vergata University, Roma, Italy; 2 Anesthesiology, Tor Vergata University, Roma, Italy

## Background

Unaortic coronary artery bypass with all arterial grafts proved to prevent early neurologic injury. Various devices for clampless proximal anastomosis of venous grafts were associated with an increased risk of microembolism. The Cardica PAS-Port is a new, fully automated device that might be able to obtain safe proximal aorto-venous connection without an increased risk of micro- or macro embolism. We evaluated early post-operative neurologic outcome in a matched population following clampless OPCAB (CCAB) or on-pump CABG.

## Methods

366 consecutive patients were submitted to isolated coronary revascularization by a single surgeon between January 2007 to June 2011. Of these, 223 were off-pump procedures. After a propensity score adjustement, 143 pairs were selected, who received either off-pump or on-pump coronary artery bypass, with no differences in any preoperative characteristic, including history of stroke or TIA (CCAB 11%; CABG 10%) and presence of significant but not critical carotid artery disease (CCAB 22%; CABG 26%). CCAB was performed either with an all-arterial approach (n=33) or by means of automated proximal anastomosis of the venous graft(s) with the Cardica PAS-Port (n=110). Neurologic injury was defined as non-reversible (NRNI: coma or stroke) or reversible (RNI: TIA or post-operative delirium necessitating specific treatment or requiring prolonged ICU stay or mechanical ventilation).

## Results

Operative mortality was 2.4% (CCAB 1.4%; CABG 3.5%; p=NS). Rate of early neurologic damage was 5.6% (CCAB 2.1% vs. CABG 9.1%; p=0.01). Incidence of NRNI was 1.4% (CCAB 0% vs. CABG 2.8%; p=0.04), while the incidence of RNI was 4.2% (CCAB 2.1% vs. CABG 6.3%; p=0.055).

## Conclusion

CCAB prevents both early post-operative RNI and NRNI. This result can be achieved not only with a totally unaortic, “all-arterial” strategy, but also with the aid of the fully automated PAS-Port device for proximal aorto-venous anastomoses.

